# MalDA, Accelerating Malaria Drug Discovery

**DOI:** 10.1016/j.pt.2021.01.009

**Published:** 2021-02-26

**Authors:** Tuo Yang, Sabine Ottilie, Eva S. Istvan, Karla P. Godinez-Macias, Amanda K. Lukens, Beatriz Baragaña, Brice Campo, Chris Walpole, Jacquin C. Niles, Kelly Chibale, Koen J. Dechering, Manuel Llinás, Marcus C.S. Lee, Nobutaka Kato, Susan Wyllie, Case W. McNamara, Francisco Javier Gamo, Jeremy Burrows, David A. Fidock, Daniel E. Goldberg, Ian H. Gilbert, Dyann F. Wirth, Elizabeth A. Winzeler, Accelerator Consortium

**Affiliations:** 1 Department of Pediatrics, School of Medicine, University of California, San Diego (UCSD), La Jolla, CA 92093, USA; 2 Department of Internal Medicine, Division of Infectious Diseases, Washington University School of Medicine, Saint Louis, MO 63130, USA; 3 Department of Molecular Microbiology, Washington University School of Medicine, Saint Louis, MO 63130, USA; 4 Bioinformatics and Systems Biology Graduate Program, University of California, San Diego (UCSD), La Jolla, CA 92093, USA; 5 Department of Immunology and Infectious Diseases, Harvard T.H. Chan School of Public Health, Boston, MA 02115, USA; 6 Infectious Disease and Microbiome Program, Broad Institute, Cambridge, MA 02142, USA; 7 Wellcome Center for Anti-Infectives Research, Division of Biological Chemistry and Drug Discovery, University of Dundee, Dundee DD1 5EH, UK; 8 Medicines for Malaria Venture, 1215 Geneva 15, Switzerland; 9 Structural Genomics Consortium, Research Institute of the McGill University Health Centre, Montreal, QC H4A 3J1, Canada; 10 Department of Biological Engineering, Massachusetts Institute of Technology (MIT), Building 56-341, 77 Massachusetts Avenue, Cambridge MA 02139-4307, USA; 11 Drug Discovery and Development Centre (H3D), University of Cape Town, Rondebosch 7701, South Africa; 12 South African Medical Research Council Drug Discovery and Development Research Unit, Department of Chemistry and Institute of Infectious Disease and Molecular Medicine, University of Cape Town, Rondebosch 7701, South Africa; 13 TropIQ Health Sciences, 6534 AT, Nijmegen, The Netherlands; 14 Department of Biochemistry and Molecular Biology and Department of Chemistry, Huck Center for Malaria Research, The Pennsylvania State University, University Park, PA 16082, USA; 15 Wellcome Sanger Institute, Wellcome Genome Campus, Hinxton, UK; 16 Global Health Drug Discovery Institute, Zhongguancun Dongsheng International Science Park, 1 North Yongtaizhuang Road, Beijing 100192, China; 17 Calibr, a division of The Scripps Research Institute, La Jolla, CA 92037, USA; 18 Tres Cantos Medicines Development Campus, Diseases of the Developing World, GlaxoSmithKline, Tres Cantos, 28760, Madrid, Spain; 19 Department of Microbiology and Immunology and Division of Infectious Diseases, Department of Medicine, Columbia University Irving Medical Center, New York, NY 10032, USA; 20 The complete author list is provided in the Acknowledgments

## Abstract

The Malaria Drug Accelerator (MalDA) is a consortium of 15 leading scientific laboratories. The aim of MalDA is to improve and accelerate the early antimalarial drug discovery process by identifying new, essential, druggable targets. In addition, it seeks to produce early lead inhibitors that may be advanced into drug candidates suitable for preclinical development and subsequent clinical testing in humans. By sharing resources, including expertise, knowledge, materials, and reagents, the consortium strives to eliminate the structural barriers often encountered in the drug discovery process. Here we discuss the mission of the consortium and its scientific achievements, including the identification of new chemically and biologically validated targets, as well as future scientific directions.

## Overview of the Malaria Drug Accelerator

For centuries, malaria has been a threat to global health and in 2019 over 400 000 people, mostly children under the age of five, died from malaria [1]. Although the use of insecticide-treated bed nets and artemisinin-based combination therapies have reduced the burden of malaria, the spread of resistance to first-line antimalarials, the loss of insecticide efficacy, and a rising number of cases highlight the need for new medicines, treatment modalities, and control measures.

Fortunately, interest in antimalarial drug development has increased over the past decade [1]. As a result, there are now at least 13 antimalarial agents in clinical development with the Medicines for Malaria Venture (MMV) and partners^i^. However, there will be attrition: some of these drug candidates will drop out during preclinical development due to, for example, lack of *in vivo* efficacy, formulation, unacceptable levels of parasite resistance, or safety issues. In addition, some compounds target only the *Plasmodium* blood stage, which causes malaria symptoms (Box 1). Therefore, there remains an important need for new antimalarial medicines, especially those that, in addition to treating *Plasmodium falciparum* malaria, also prevent transmission (to the human or mosquito vector) or provide a radical cure in the case of infection with *Plasmodium vivax* or *Plasmodium ovale* malaria, or work prophylactically.

**Figure 1.**
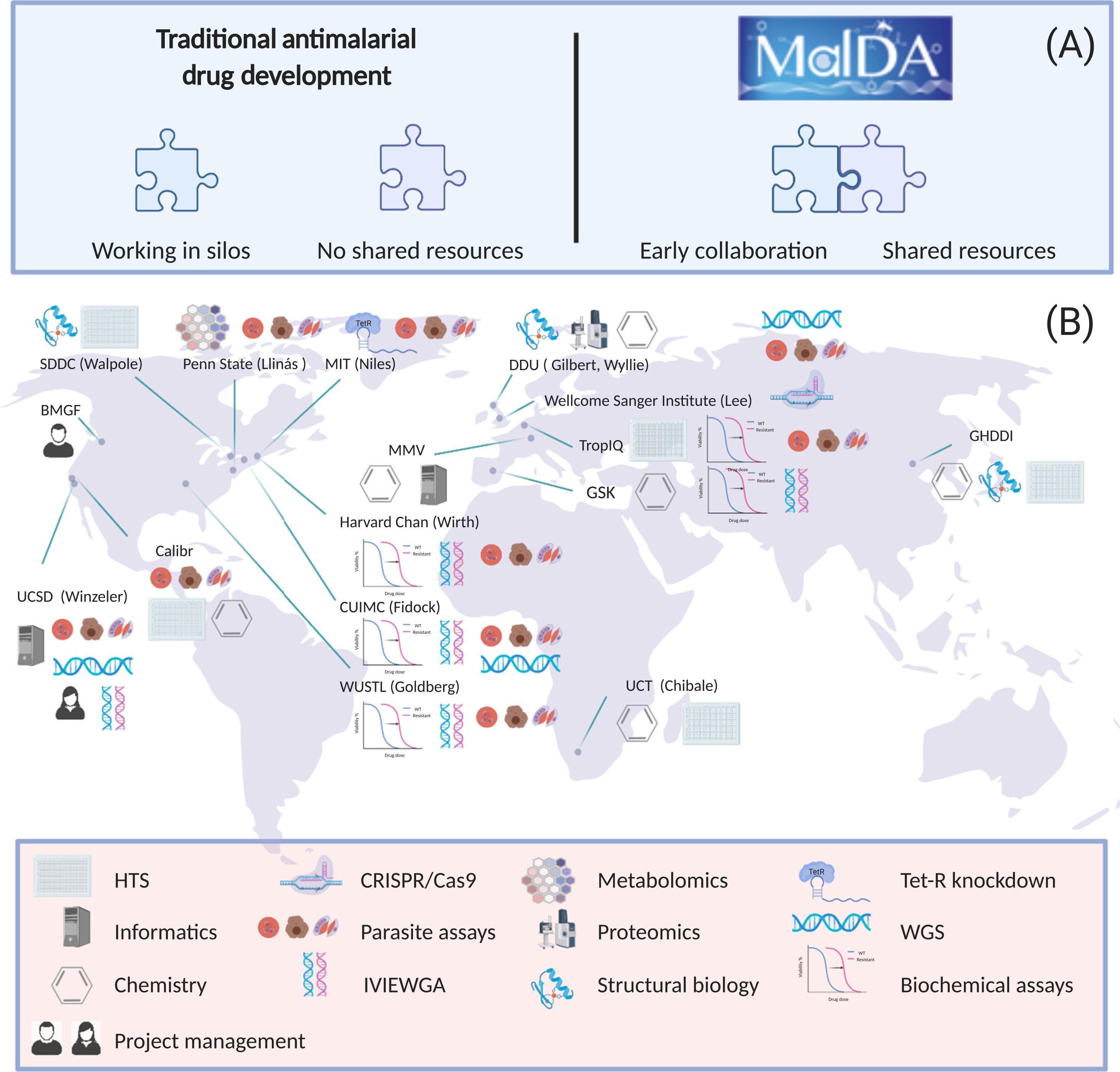
Overview of MalDA (Malaria Drug Accelerator). MalDA is a consortium of 15 scientific laboratories (B), working in the earliest stages of drug discovery. By collaborating and sharing compounds and resources, MalDA can achieve milestones quickly and efficiently (A). Abbreviations: BMGF, Bill and Melinda Gates Foundation; Calibr, California Institute for Biomedical Research; CUIMC, Columbia University Irving Medical Center; DDU, Drug discovery unit – University of Dundee; GHDDI, Global Health Drug Discovery Institute; GSK, GlaxoSmithKline; Harvard Chan, Harvard T.H. Chan School of Public Health; HTS, High-throughput screening; IVIEWGA, *in vitro* evolution and whole-genome analysis; MIT, Massachusetts Institute of Technology; MMV, Medicines for Malaria Venture; Penn State, The Pennsylvania State University; SDDC, Structure-guided Drug Discovery Coalition; TropIQ, TropIQ Health Sciences; UCSD, University of California San Diego; UCT, University of Cape Town; WGS, Whole-genome sequencing; WUSTL, Washington University in St Louis.

**Figure 2.**
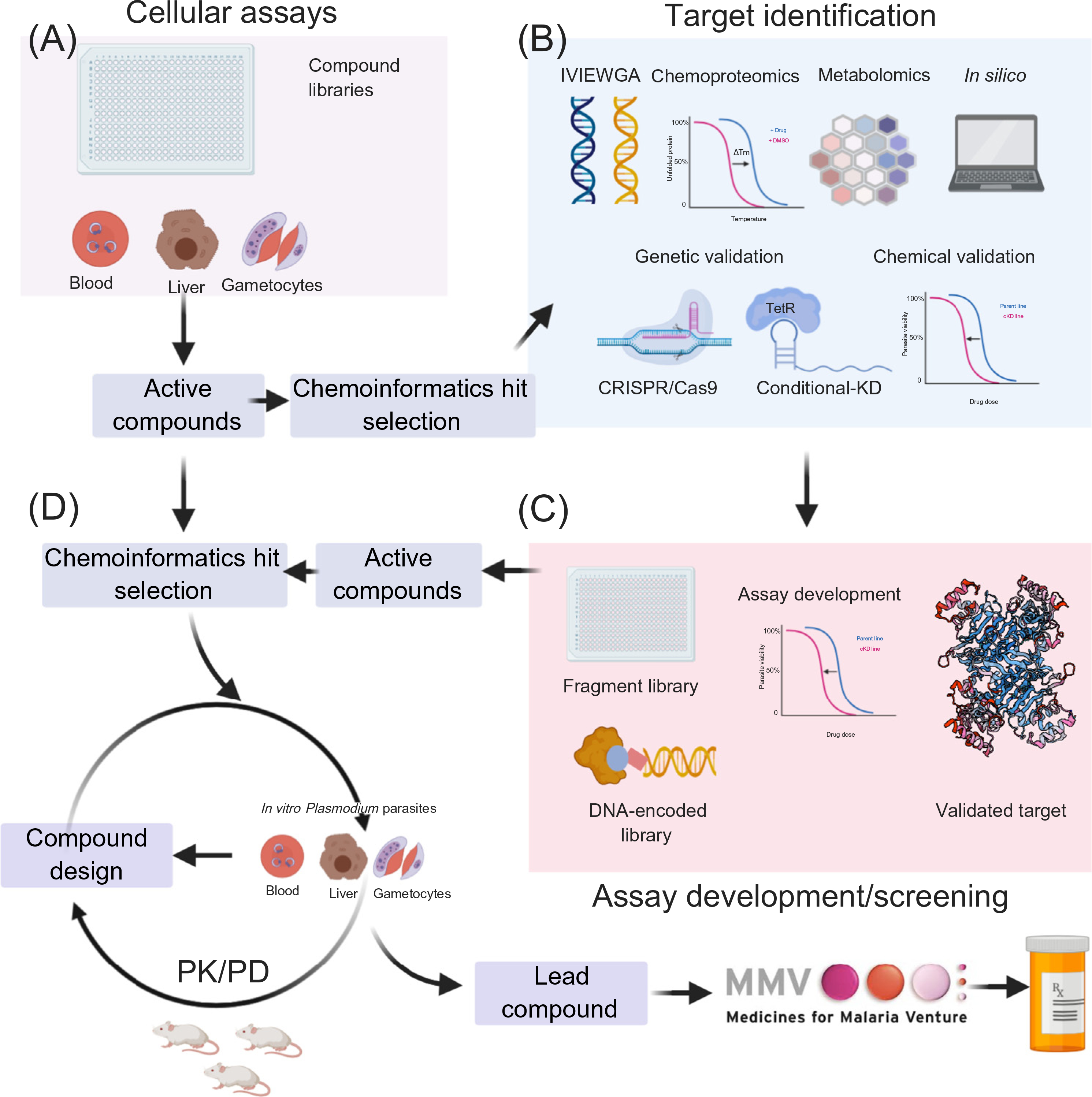
Overview of MalDA (Malaria Drug Accelerator) Pipeline for the Discovery of Novel Antimalarials. Phenotypic drug discovery includes screening compound libraries against blood- and liver-stage *Plasmodium* parasites (A). The first steps to identify the target of an active hit compound utilize *in vitro* evolution and whole-genome analysis (IVIEWGA), metabolomics, chemoproteomics, and chemoinformatics approaches, followed by target validation via genetic and chemical approaches (B). Genetic validation uses clustered regularly interspaced short palindromic repeats/Cas9 (CRISPR/Cas9) to reconstitute the resistant alleles found in IVIEWGA experiments. In conditional knockdown (KD) experiments, parasites are subjected to compound challenges to probe for dose–response changes (chemical validation and vulnerability assessment) and confirm compound hypersensitivity through diminished target expression (B). Validated targets can be rescreened for additional specific inhibitors using, for example, DNA-encoded libraries or target-specific libraries (C). Active compounds from phenotypic screening assays can also be directed to hit-to-lead development to be optimized for enhanced pharmaceutical properties. The resulting lead compounds are further examined in the *in vitro* assays against different malaria parasite developmental cycles and *in vivo* efficacy and pharmacokinetic/pharmacodynamics (PK/PD) studies in *Plasmodium falciparum* severe combined immunodeficiency (SCID) or NSG mouse models (D).

Recognizing that private investment in malaria research is likely to be limited, the Malaria Drug Accelerator (MalDA) was formed in 2012. Currently, MalDA is a consortium of 15 scientific laboratories (Figure 1) whose contributions are funded by the Bill and Melinda Gates Foundation (BMGF). The goal of MalDA is to accelerate the development of new therapies for malaria by investing in new target discovery, screening, and hit-to-lead development. The objective of the consortium is to use resources collaboratively by sharing reagents, materials, and expertise, thus preventing ‘siloing’ of projects or knowledge and improving efficiency (Figure 2).

## History of Antimalarial Drug Discovery

To understand MalDA, it is important to know the history of antimalarial drug discovery work over the past century. Initially, natural products, such as quinine and artemisinin (see the review in [2]), were used in malaria treatment. Further, semisynthetic artemisinin derivatives were developed to improve the poor biopharmaceutical properties of artemisinin. However, resistance to this drug emerged as a result of noncompliance with the 3-day regimen, artemisinin’s short half-life, and parasite recrudescence. This, in turn, limits artemisinin’s therapeutic effects [3]. In response to this issue, fully synthetic antimalarials, such as OZ277 and OZ439 were developed with improved pharmacokinetics aiming to overcome artemisinin resistance. These are often based on chemical scaffolds of older natural products. For example, OZ277 and OZ439 incorporated the endoperoxide bridge of artemisinin [4,5]; OZ277 is now marketed as Synriam (in combination with piperaquine) [6], and OZ439 is in clinical trials [7].

Over the past 15 years, antimalarial drug candidates have been more typically discovered using phenotypic screening approaches [8–10]. More than seven million compounds have now been tested against asexual blood-stage parasites. To identify inhibitors targeting other parasite life cycle stages, over 500 000 compounds were recently screened against murine liver-stage parasites, resulting in the identification of 681 validated hits effective against hepatic schizonts [target candidate profile (TCP 4)] at submicromolar concentrations [11] (Box 1). In an independent screen of 70 000 chemical compounds, 17 were identified having transmission-blocking activity (TCP 5) [12] (Box 1). Over the past decade, some of these active screening hits have been further profiled and optimized through rounds of medicinal chemistry aimed at identifying a nontoxic clinical candidate with an acceptable predicted efficacious oral dose in humans; such candidates could progress to human clinical trials following preclinical good laboratory practice (GLP) safety studies.

Examples of drug candidates that originated from phenotypic screens include KAE609 (cipargamin) and KAF156 (ganaplacide). Both are effective against *Plasmodium* parasites at nanomolar concentrations [13]. The latter is a novel imidazolopiperazine-based antimalarial that is pan-active against *Plasmodium* parasites [13,14]. This compound exerts its function by inhibiting protein trafficking and causes endoplasmic reticulum (ER) expansion in parasites, though its specific target is unknown [15]. It has good pharmacokinetic properties and is orally available [16]. Given its blood-stage antimalarial potency as well as transmission-blocking activity, KAF156 could play an important role in eliminating malaria. KAF156 is now undergoing Phase IIb clinical trials (NCT03167242) in combination with lumefantrine.

A limitation of phenotypic screens is that, in the absence of biochemical or cellular target deconvolution assays, there is no information regarding the mechanism of action. Knowing the target is important for compound optimization and safety evaluation. First, without knowing which part of the small molecule interacts with its protein target it can be difficult to predict which chemical changes will result in improved binding and thus improved efficacy. Second, ignorance of the biological target can hinder optimization of the chemical series against mammalian or human orthologs that could, in later stages, preclude development. The amount of chemistry that has been explored through phenotypic screening in the pursuit of potent ‘hits’ is so extensive that finding novel compound libraries to screen is becoming increasingly difficult. Despite these challenges, as mentioned earlier, numerous successful candidates from phenotypic screens have been identified and are candidates for further development.

## Target-Based Drug Discovery

A more elegant approach to drug discovery is target-based discovery. In this case, a recombinant, chemically validated target is used in biochemical screening. Rational drug design, wherein chemists predict which changes should be made to the drug candidate to optimize potency based on the target-compound interaction, is also possible. Computational approaches facilitate visualization of the interaction between a compound and its target, thereby guiding the structure–activity relationship (SAR) (see Glossary). Where it is possible to cocrystallize the enzyme, this allows structure-based and fragment-based drug discovery approaches.

In addition, target-based screening, even without a structure, greatly facilitates drug discovery. It allows counter-screens against the human homolog to optimize for selectivity. It also allows for approaches such as DNA-encoded library (DEL) technology. Here, billions of different compounds, each attached to a DNA barcode, can be incubated with a target in a single test tube [17]. The approach requires a small amount of target protein (at μg scale) so that multiple conditions can be tested at once in an efficient manner, together with functional assays, inhibitors can be identified and characterized. The work is not dependent on having parasites, which require substantial effort to culture and maintain. Importantly, it can be extended to biological targets from *Plasmodium* species refractory to laboratory *in vitro* culture. DEL technology has been used to discover compounds inhibiting human receptor interacting protein 1 kinase (RIP1K) for inflammatory disease [18], and some derivatives are now in clinical trials [19].

Target-based drug discovery is not entirely new for malaria, and several antimalarial drug candidates that were identified this way have progressed to human trials, including molecules that target dihydroorotate dehydrogenase (DHODH) and dihydrofolate reductase (DHFR) [20–22]. However, the critical problem has been the dearth of well-validated antiplasmodial targets.

To address the need for additional targets, in the first several years of its existence MalDA was focused on the discovery and validation of novel targets (see the review in [23]) that could eventually be used in target-based drug discovery. Although functional genomic methods have been used to identify proteins that are essential to parasite viability, and might be attractive targets for intervention [24,25], chemical and genetic validation are fundamentally different. Genetic validation means that the gene encoding the protein target is needed for parasite survival. However, gene essentiality does not mean that the protein is druggable. To be druggable, the protein target needs to have a binding site for a small molecule that has potential for oral absorption, and it needs to be expressed at the right time of the parasite’s life cycle. Furthermore, it needs to be essential such that even partial inhibition with a small molecule results in inhibition of parasite growth and ultimately parasite death. Such targets are described as ‘chemically validated’ (Box 2). Examples of chemically validated targets are given in Table 1.

## IVIEWGA: *In Vitro* Evolution and Whole-Genome Analysis

A very successful method for identifying chemically validated targets is through *in vitro* evolution and whole-genome analysis (IVIEWGA), a reverse-genetic method that takes compounds with activity in phenotypic screens and then identifies the target. Because it is already known that treatment with a compound will kill the parasite, it is expected that targets revealed with this approach will be chemically validated.

The first step of IVIEWGA is to expose blood-stage parasites to sublethal concentrations of a compound in anticipation that a few parasites will eventually develop resistance via mutations in the target. As malaria parasites have haploid genomes, for some compounds only a single mutation in one gene might be needed to acquire resistance. The process may take several months and is typically a function of the intrinsic mutation rate per base pair, the starting number of parasites and replication cycles used during the selection process, the number of pathways and steps to resistance, and the type of gene mutation(s) that confers resistance (e.g., mitochondrial genes, such as cytochrome bc1, are present at higher copy in the cell, leading to more chances for mutation [26]).

## Shared Bioinformatic Protocols Improves Efficiency

The next challenge in IVIEWGA is to identify the mutations that emerge during long-term culture. Due to the difficulties of performing genetic crosses of *P. falciparum*, whole-genome sequencing and comparative genomics between wild-type and resistant clones is now widely utilized to identify resistance-causing genetic changes – single-nucleotide variants (SNVs) and copy number variations (CNVs). Both the evolved resistant clone and the parent clone are sequenced to at least ~50× average genome coverage. Depending on the time required to select a resistant line, sequencing may reveal many possible allelic changes in an evolved clone [27]. The number of mutations in drug-pressured parasites can be higher when using *P. falciparum* lines with mutations in DNA polymerase δ, which appears to confer an increased mutation rate under drug pressure [28]. However, mutations that confer resistance have specific characteristics: the allele fraction is typically 100% for alleles that confer resistance, unless the allelic change is within a CNV; a resistance-conferring change is generally in the core genome [29,30] and not in one of the genes associated with antigenic variation or at a chromosome end; the allele is not present in the parent, and the mutation leads to a change in the protein-coding sequence. Furthermore, the allelic change often maps to a conserved part of the predicted protein, sometimes in a small-molecule binding site (e.g., an ATP-binding site). Because there may be about three SNVs per evolved line that satisfy these criteria, multiple independent clones are often examined, thereby providing increased confidence in the identification of causative alleles. CNVs can also be identified. In some cases, SNVs are found within CNVs [29,31,32]. Standardization of the analysis pipeline and a database with sequences of hundreds of evolved clones further allow candidate mutations to be more readily identified by the MalDA consortium. The majority of the targets in Table 1 were either discovered or rediscovered using IVIEWGA.

## A CRISPR/Cas9 Pipeline

Because mutations may randomly arise during long term-culturing, SNVs discovered in experimental evolution cell lines require validation. One method of genetic validation is to perform an allelic replacement by the site-specific introduction of a mutant allele into a naïve genetic background, that is, a parasite clone that was not subjected to drug exposure and *in vitro* evolution. Although allelic replacement methods have been used in the past [32], recent developments in gene editing methodologies are rapidly advancing the abilities of researchers to probe gene functions in malaria parasites [33–35]. Using clustered regularly interspaced short palindromic repeats/Cas9 (CRISPR/Cas9), it is possible to target double-strand breaks to specific sequences using guide (g)RNAs to introduce desired SNVs with the aid of short (~500 nt) donor-repair sequences. This approach is also valuable for dissecting more complex situations in which multiple mutations may contribute to the observed resistance phenotype.

The next task is to determine whether the resistance-conferring mutation is in the actual target or in a more general resistance gene, such as *pfmdr1*. For further target validation, a tunable translational repression system has been particularly helpful [36,37]. Additionally, dose–response curves of conditionally expressed proteins (cKD) for compounds that putatively interact with a specified target protein can be determined at various protein expression levels to determine whether hypersensitization occurs (as evidenced by a decrease in EC_50_). Target validation can be particularly convincing if the phenotype of inhibition by compound aligns with the phenotype of the knockdown as described in [38–40]. Use of this cKD approach in validating a variety of putative targets allows for a standardized method to compare the druggability of targets. This conditional expression system can be used more generally to identify structurally different compounds engaging the same target, as the effects of SNVs on EC_50_ will depend on whether scaffolds share the same target binding or interacting sites.

## Examples: *In Vitro* Evolution Has Proven to Be a Successful Approach for Target Discovery by the MalDA Consortium

### *Pf*NCR1

Very recently, an uncharacterized parasite protein, *P. falciparum* Niemann–Pick type C1-related protein (*Pf*NCR1), was first identified by selecting parasites against three diverse antimalarial compounds using IVIEWGA [38]. *Pf*NCR1 acquired SNVs or CNVs after selections using these three compounds. Further investigation showed that *Pf*NCR1 localizes to the parasite plasma membrane and is required for digestive vacuole biogenesis [38]. The essentiality of this gene in parasite asexual growth, as well as its other druggable features, highlight the possibility that *Pf*NCR1 could be a novel drug target.

### aaRSs

Aminoacyl-tRNA synthetases (aaRSs) play a crucial role in protein biosynthesis pathways. *Plasmodium* parasites have 36 aaRS enzymes, and given their important functions, targeting *Plasmodium* aaRSs provides a new resource of targets for antimalarial drug development. So far, many *Pf*-aaRSs have been characterized, such as the arginyl- [41], tryptophanyl- [42,43], isoleucyl- [32], prolyl- [44], and tyrosyl- [45] aaRSs. These studies provide a basis for future inhibitor design and screening strategies. A pan-active bicyclic azetidine, BRD3444, was identified from phenotypic screening [46]. *In vitro* evolution using BRD1095, a derivate of BRD3444 with enhanced solubility, identified SNVs in the phenylalanyl-tRNA synthetase (*Pf*PheRS) [46]. Follow-up *in vitro* enzyme activity assays confirmed that *Pf*PheRS is the target of bicyclic azetidines.

### Acetyl-CoA Synthetase

Acetyl-CoA is a key molecule in cellular metabolism and regulation of cellular acetyl-CoA levels and is essential for cell survival. Acetyl-CoA is required for the tricarboxylic acid (TCA) cycle, lipid and phospholipid synthesis, and for histone acetylation, a key regulatory mechanism for gene expression. IVIEWGA identified point mutations in the *P. falciparum* acetyl-CoA synthetase gene, implicating this enzyme as a putative drug target, and metabolomic profiling pointed to changes in parasite acetyl-CoA levels following drug exposure [47,48]. Subsequent allelic replacement using CRISPR/Cas9 technology, conditional expression modulation, and enzymatic analysis of heterologous expressed protein confirm that acetyl-CoA synthetase is the target of these molecules and that this enzyme is essential for parasite survival [48]. Drugs developed targeting acetyl-CoA synthetase would have dual action in both the liver and blood stages of infection and thus be useful in both prevention and treatment.

## Other Approaches to Target Identification

For compounds where resistance cannot be achieved, or that are associated with mutations in general drug-resistance genes rather than their cellular targets, it can be difficult to identify targets using IVIEWGA alone. As such, other approaches are employed to elucidate their targets or pathways.

## Metabolomic Profiling

Metabolomic profiling measures changes in the parasite’s metabolites after compound exposure and in some cases can generate a recognizable ‘fingerprint’ resulting from perturbations in specific metabolic pathways [27,49,50]. Compounds that act against pyrimidine biosynthesis in the parasite mitochondrion typically have a distinct metabolic profile, and metabolic profiling was used to correctly predict the mode of action (MOA) of compounds with liver-stage activity [11]. A recent study led by MalDA collaborators generated metabolic fingerprints or MetaPrints at different asexual stages of *P. falciparum* following compound treatments [50]. Metabolomic profiling, combined with stage-specific assays, may also provide insights into the mode of action of a wide array of antimalarial molecules [51,52]. This approach works best when comparative MetaPrints for reference inhibitors with similar mechanism of action are available (e.g., with atovaquone, an inhibitor of pyrimidine biosynthesis).

## Chemoproteomics

Chemical proteomics approaches provide alternative routes to identify the molecular target(s) of phenotypically active compounds. Importantly, these approaches often provide direct evidence of on-target engagement not provided by the other methodologies employed by the consortium. One such strategy is to immobilize active compounds onto magnetic beads, with these drug beads then being used to pull-down interacting proteins from parasite cell extracts. The discrimination of proteins that bind specifically/nonspecifically to beads is improved by carrying out these pull-down experiments in the presence/absence of competing free compound. Proteins binding specifically to drug beads can then be identified and quantified via mass spectrometry (MS) and identified as putative drug targets. Indeed, such an approach was used successfully to confirm that 2-aminopyrazine MMV390048 and its analogs specifically target phosphatidylinositol 4-kinase (PI4K) in *P. falciparum* [53]. Although PI4K was also discovered by IVIEWGA with the same compound, in these elegant studies chemoproteomics was also used to interrogate and rule out potential ‘off-target’ human cell interactions that may represent a toxic liability for the development of this promising antimalarial series. Another successful example using chemoproteomics is the identification of *P. falciparum* cGMP-dependent protein kinase (*Pf*PKG) as the primary target of a pan-active trisubstituted imidazole, MMV030084 [40]. IVIEWGA yielded mutations in tyrosine kinase-like protein 3 (TKL3), a resistance mediator for PKG inhibitors (e.g., MMV030084), but no mutations in *Pf*PKG. Combined metabolomics, phosphoproteomics, chemoproteomics, and genetics identified and confirmed PKG as the target of MMV030084 [40].

In cases where immobilization of compounds onto beads compromises the ability of the compound to bind to its target, or if there is limited or no SAR data available, then label-free proteomic approaches, such as cellular thermal-shift assay (CETSA) or thermal proteomic profiling (TPP) can be utilized. Both methods are based on the principle that the binding of a drug to its protein target can significantly increase the thermal stability of that protein. The thermal stability of proteins within a parasite cell lysate can be monitored in the presence or absence of the test compound using quantitative MS enabled by tandem mass tags (TMTs). This approach has recently been applied to interrogate the target of quinine [54]. Dziekan and colleagues exposed *P. falciparum* lysates to different quinine concentrations at 51°C to achieve high proteome coverage [54]. Quantitative MS provided evidence that *P. falciparum* purine nucleoside phosphorylase (*Pf*PNP) binds to quinine and is stabilized at 100 nM quinine concentration. Interaction of quinine and *Pf*PNP was further supported by crystallography [54]. In common with other chemical proteomics strategies, TPP can be used to provide direct evidence of on-target engagement and can be used at multiple stages of the drug discovery process. Chemoproteomics is being employed to identify the molecular targets of a number of compounds of interest to the MalDA. However, just because a protein is stabilized by a compound, or interacts with a protein based on crystallography studies, does not mean that the protein is the compound’s true or only target.

## *In Silico* Approaches

In some cases, targets can be discovered via chemoinformatics. Various phenotypic screening campaigns have identified more than 30 000 compounds with antimalarial activities [11,55]. If a compound under consideration shows structural similarity to compounds with known targets, then specific hypotheses about function can result. MalDA researchers have applied cluster analysis to successfully identify scaffold families from the Tres Cantos antimalarial set (TCAMS), the Charles River library, or other compound collections [9,11]. Follow-up drug activity assays validated the results from the clustering analysis. Additionally, scaffold analysis revealed novel antimalarial molecules with dual-stage activity, as shown using the chemically diverse Global Health Chemical Diversity Library [55].

*In silico* approaches allow researchers to not only predict a specific drug–target interaction but also provide the opportunity to predict the biological impact of a compound. To achieve this, chemoinformatic pipelines have been developed. As an example, the open-source MAIP system [56] illustrates the use of machine learning to predict compounds with possible antimalarial properties. It is noteworthy to mention that, while these tools provide guidelines for drug-target identification or parasitological efficacy, follow-up validation is crucial. *In silico* methods nonetheless reduce experimentation time, resources, and efforts.

## Reverse Genetic Approaches to Target Discovery

Evidence of genetic essentiality and a hypothesis can also lead to the prioritization of targets. For example, *P. falciparum* blood-stage parasites require host glucose for energy, and the *P. falciparum* hexose transporter 1 (*Pf*HT1) can transport both glucose and fructose whereas host glucose transporter 1 (GLUT1) is selective for glucose. Recent availability of the *Pf*HT1 crystal structure enabled observation that C3361 binding induces conformational rearrangement of *Pf*HT1, and C3361 derivatives with improved selectivity for *Pf*HT1 over human GLUT1 were described [57,58]. Together, these findings make PfHT1 an attractive antimalarial target with the promise of using target-based, structure-guided approaches to develop selective inhibitors of *Plasmodium* glucose transport.

## Irresistible and Target-less Compounds

Even though *in vitro* evolution often yields resistant parasites, these attempts can fail. Compounds with this outcome have been termed ‘irresistible’ [59]. Irresistible compounds are attractive starting points for drug development as they are less likely to result in clinical resistance and therefore remain efficacious for a long time. There are many factors that could potentially contribute to drug resistance, such as copy numbers of a target, ways to generate resistance, and parasite fitness. However, to our knowledge, no studies have been performed systematically to compare different resistance factors. To help predict resistance liability, resistance potential can be quantified as the minimum inoculum for resistance (MIR) [60] and, in turn, inform drug development prioritization.

In some cases, resistant parasites can be obtained through *in vitro* evolution experiments; however, the targets cannot be elucidated. We categorize compounds that only yield mutations in multidrug-resistance genes (MDR genes, such as *pfmdr1* or *pfcrt*), as target-less compounds if this is the only information available. *P. falciparum* parasites acquire CNVs and SNVs in MDR genes frequently after challenge with different compounds. For example, Cowell *et al.* showed that *pfmdr1* mutations arose for six of 37 different compounds [27]. Other genes that were seen frequently included *pfcrt* and *pfaat1* [27].

KAF156 is one such target-less compound. IVIEWGA of the imidazolopiperazine KAF156 showed SNVs in *pfcarl*; however, mutations in this essential gene also lead to increased EC_50_ to compounds that are structurally unrelated to KAF156, suggesting that this protein, most likely, mediates resistance to KAF156 as well as other diverse scaffolds [61–63]. Additionally, 4- and 8-animoquinolines, the arylamino alcohols, and artemisinins may also be considered target-less. A combination of approaches can be applied to identify targets for compounds for which using a single strategy proved insufficient [15].

## Next Steps: Structure Determination and Progression through the Drug Discovery Process

Once a target has been identified and validated, concerted efforts are made to determine the structure (if unknown) as well as produce recombinant protein for target-based screening. Structure determination is largely performed by MalDA consortium members, the Structure-guided Drug Discovery Coalition^ii^, and the Structural Genomics Consortium (SGC)^iii^, but may also be undertaken by individual laboratories. Following triaging and validation of hits from the aforementioned target-based screening, multiple chemical series can be progressed through the drug discovery process, underpinned by medicinal chemistry integrated with preclinical pharmacology, to deliver quality novel leads whose characteristics are defined by the MMV Target Candidate Profile (TCP; Box 1) criteria for early leads.

*P. falciparum* cytosolic lysyl-tRNA (*Pf*KRS1) is a good example of how targets can advance. *Pf*KRS1 was identified as the target of cladosporin using a variety of target deconvolution methods [64]. Even though cladosporin inhibits *Pf*KRS1 and shows potent activity against *Pf*3D7 parasites (with EC_50_ = 73 nM), cladosporin cannot be developed as an antimalarial drug due to its metabolic instability. Nevertheless, *Pf*KRS1, an aaRS (see earlier), is essential in different parasite life cycle stages and can be selectively inhibited (some compounds show >100-fold lower activity against the human enzyme, making it an attractive target [64]). To identify novel *Pf*KRS1 inhibitors and overcome cladosporin liabilities, a crystal structure was determined and the recombinant protein used to screen ~13 000 compounds [65]. A promising hit was identified and structure-guided medicinal chemistry efforts resulted in a lead compound with improved stability [65]. The lead shows excellent oral bioavailability, and reduced parasitemia by 90% in a severe combined immunodeficiency (SCID) mouse model of *P. falciparum* infection [65].

The proteasome plays an important role in degrading ubiquitinated proteins to maintain cellular protein homeostasis [66], and the 20S proteasome is the active core for protein degradation. The proteasome has been an attractive antimalarial drug target due to its involvement in maintaining proteostasis across the whole life cycle. Many proteasome inhibitors have demonstrated good activity against malaria parasites; however, as the proteasome is conserved across eukaryotes, these inhibitors can be toxic to human cells [67]. MS and substrate profiling have been performed to identify proteasome active site preferences in order to improve inhibitor specificities against malaria parasites [68,69]. As a result, the highly selective vinyl sulfones, WLW-vs and WLL-vs, were synthesized [68]. *In vitro* studies showed that these inhibitors synergized with artemisinin to enhance clearance of artemisinin-resistant parasites including in mouse models of malaria infection [68,70]. The crystal structure of the *Pf*20S core proteasome with its regulatory *Pf*PA28 cap is now available and this will provide more structure–function insights into the proteasome that should facilitate improved inhibitor designs [71].

## Concluding Remarks

The MalDA consortium provides an excellent model for collaboration in drug discovery, especially for rare or neglected diseases. The tuberculosis (TB) Drug Accelerator Program (TBDA) is another successful collaboration among academic and industry laboratories to speed up the discovery and development of novel compounds active against TB [72]. In both cases, drug development may be constrained by limited resources, expertise, and funding. Cooperation can also lessen the waste of precious resources and knowledge.

MalDA provides a unique forum to accelerate malaria target-based drug discovery. By sharing resources and expertise among academic and industry partnerships – for example, compounds that have been selected by MalDA members for further investigation are not only from MMV libraries but also from outside collaborators – so far, more than 200 compounds are under investigation for target identification, and at least 23 putative targets have been identified by the MalDA consortium. Even though questions and obstacles still exist for malaria drug discovery (see Outstanding Questions), MalDA has built on the advances of parasite biology and has the ability to validate, express, and screen new malaria targets, resulting in a complementary hit and lead generation paradigm to deliver into the MMV Discovery portfolio.

## Figures and Tables

**Table 1.** Select Chemically Validated Drug Targets

Drug target	Class^a^	Inhibitors	Refs
Dihydroorotate dehydrogenase (DHODH) (PF3D7_0603300)	VT4	DMS1; DSM74; DSM265; Genz-669178; Genz-666136; BRD9185	[20,46,74–78]
Cytochrome b1 (mal_mito_3)	VT4	Atovaquone; decoquinate; tetracyclic benzothiazepine; ML238; BRD6323; ELQ300; GW844520; GSK932121	[79–83]
PfATP4 (PF3D7_1211900)	VT4	KAE609; GNF-Pf-4492; PA21A092; SJ733	[84–87]
1-deoxy-D-xylulose 5-phosphate reductoisomerase (DXR) (PF3D7_1477300)	VT4	Fosmidomycin	[88]
Dihydrofolate reductase-thymidylate synthetase (DHFR-TS) (PF3D7_0417200)	VT4	MMV027634, P218, pyrimethamine; cycloguanil	[21,27,89–92]
Phosphatidylinositol 4-kinase (PI4K) (PF3D7_0509800)	VT4	KDU691; MMV390048; KAI407; BQR695; BRD73842	[46,53,93]
Translation elongation factor2 (eEF2) (PF3D7_1451100)	VT3	DDD107498	[94]
Farnesyl/geranylgeranyl diphosphate synthetase (fpps/ggpps) (PF3D7_1128400)	VT3	MMV019313	[95–99]
N-Myristoyltransferase (NMT) (PF3D7_1412800)	VT3	IMP-1002; DDD85646	[100]
Hexose transporter 1 (HT) (PF3D7_0204700)	VT3	Compound 3361	[57]
Farnesyltransferase (PF3D7_1147500)	VT3	BMS-388891; MMV019066	[27,101,102]
Cytoplasmic prolyl-tRNA synthetase (cPRS) (PF3D7_1213800)	VT3	Halofuginone	[44,103,104]
Isoleucyl-tRNA synthetase (cIRS) (PF3D7_1332900)	VT3	Thiaisoleucine	[32]
Plasmepsin X, IX, and V (PF3D7_0808200, PF3D7_1430200, PF3D7_1323500)	VT3	WM382; WEHI 842	[105,106]
Cytosolic lysyl-tRNA synthetase (PfKRS1) (PF3D7_1350100)	VT3	Cladosporin; Compound 5	[64,65]
cGMP-dependent protein kinase (PKG) (PF3D7_1436600)	VT3	ML10; MMV030084	[40,107]
Phenylalanyl-tRNA synthetase [35] (PF3D7_0109800) (PF3D7_1104000)	VT3	BRD1095	[46]
Proteasome subunit beta-5 (PF3D7_1011400)	VT3	Bortezomib; Carfilzomib; Carmaphycin B; WLL-vs	[67–69]
Cyclin-dependent-like kinase 3 (CLK3) (PF3D7_1114700)	VT3	TCMDC-135051	[108]
Niemann–Pick type C1-related protein (NCR1) (PF3D7_0107500)	VT3	MMV009108; MMV019662; MMV028038	[38]
Acetyl-CoA synthetase (AcAS) (PF3D7_0627800)	VT3	MMV689258; MMV019721; MMV084978	[47,48]
Purine nucleoside phosphorylase (PNP) (PF3D7_0513300)	VT3	DADMe-ImmG;	[109]
Cleavage and polyadenylation specificity factor subunit3 (CPSF) (PF3D7_1438500)	VT3	Benzoxaborole AN3661	[110]

a See Box 2 for the criteria of validated target (VT) classes.

## Box 1. *Plasmodium* Life Cycle and Points for Intervention

Malaria is a general term for a febrile infection caused by *Plasmodium* parasites, and may be caused by at least five different species of the parasite, each of which may show differential sensitivity to drugs and which have different drug-resistance profiles. *P. falciparum* is the deadliest species, whereas *P. vivax* accounts for malaria relapse, making it a major challenge to malaria eradication. The *Plasmodium* parasite life cycle involves the *Anopheles* mosquito as vector and a vertebrate secondary host. In the human host, the parasite undergoes exoerythrocytic (liver-stage) and intraerythrocytic asexual development. Activation of dormant *P. vivax* hypnozoites in the liver causes malaria relapse. The intraerythrocytic asexual division of *Plasmodium* parasites causes the clinical symptoms of malaria. During each intraerythrocytic asexual cycle, a subpopulation of parasites commits to sexual development into gametocytes. Mature gametocytes are ready to be taken up by mosquitoes; hence the gametocyte stage is crucial for malaria transmission (Figure I).

While many antimalarial compounds target the pathogenic asexual blood stages, ideally, new antimalarials should also target other stages. Target candidate profiles (TCPs) were proposed in 2013 as strategic tools to guide malaria drug discovery. Currently, there are five TCPs [73] (Figure I):

TCP1: Molecules that clear asexual blood-stage parasitemia – blood-stage treatment.

TCP3: Molecules with activity against hypnozoites – hypnozoite antirelapse treatment.

TCP4: Molecules with activity against hepatic schizonts – liver prophylaxis.

TCP5: Molecules that block transmission (targeting parasite gametocytes) – gametocyte transmission blocking.

TCP6: Molecules that block transmission (targeting the insect vector) – mosquitocidals.

## Box 2. Classes of Validated Antimalarial Target

Through the collaborative efforts of MalDA and many other scientific laboratories, many new targets have been discovered and validated. Since the drug development process requires numerous steps, many compounds inhibiting genetically and chemically validated targets still need further evaluation before progressing into lead development. For example, the resistance potential is an important factor to consider when developing drugs. Structural information is needed for structure-based and fragment-based drug design; toxicity and druggability are key to the design of drug-like inhibitors.

We propose several validated target (VT) classes based on the criteria below. These criteria could be used as a guideline for future drug development.

VT1 – Genetically validated target. Gene encoding the target demonstrates essentiality from genetic modification assays.

VT2 – Chemically validated target. Inhibition demonstrated with tool compounds.

VT3 – All of the above criteria plus a known resistance potential. This resistance potential can be determined with known mutations (gene amplifications) in wild-type parasite populations from *in vitro* evolution assays.

VT4 – Clinically validated targets. Compounds acting against these targets eliminate symptoms of malaria in humans. Examples include cytochrome bc1, DHODH, and DHFR (Table 1).

## Highlights

Novel antimalarial therapies are required to combat drug-resistant *Plasmodium falciparum (Pf)* and help with malaria elimination.

To address the need for antimalarial drugs, Malaria Drug Accelerator (MalDA), a public–private partnership, was formed in 2012. Today, it is a consortium of 15 academic and industrial laboratories whose collective expertise and resources have been brought together to accelerate malaria drug development.

Over the last decade, MalDA has developed a collaborative pipeline, applying multiple biomedical, biotechnological, and cheminformatic approaches to the identification of novel drug targets.

Due to the significant progress in identifying novel validated targets, advanced target-based drug discovery is underway. Chemists are performing hit-to-lead compound optimization on inhibitors of many chemically validated targets, including *Pf* phenylalanyl-tRNA synthetase *(*PheRS), *Pf* acetyl-CoA synthetase (AcAS), and *Pf* Niemann–Pick type C1-related protein (NCR1).

## Outstanding Questions

Have phenotypic screening approaches reached target saturation, and are new screening methods and chemical matter needed to find new targets? Should more natural product libraries be screened?

Are there any host genes that can be safely targeted? Can traditional screening technologies uncover compounds targeting host determinants of parasite survival?

Will thermal shift proteomic methods live up to their promise or do they work better for identifying the same cytoplasmic targets that are readily identified using *in vitro* evolution?

Can structure-guided drug discovery and artificial intelligence (AI) deliver better leads and more potent drug candidates? Will such delivery be cheaper and quicker?

Is the emergence of drug resistance driven by the chemical scaffold or the target? For example, resistance to pyrimethamine is due to mutations in its target, *Pf*DHFR. To overcome this, compound P218 was identified and optimized to bind flexibly into the *Pf*DHFR active site such that P218 showed good antimalarial activity against both the DHFR mutant and wild-type *P. falciparum* parasites, thus it could potentially be a replacement for pyrimethamine. Can resistance be predicted and circumvented?

Can clues about a compound’s intracellular site of action be obtained by analyzing the patterns of resistance mutations (e.g., mutations in proteins located at the food vacuole, mitochondria, cytosol, etc.)?

How do we find targets for liver stages or transmission-blocking drugs?

## Resources

i www.mmv.org/research-development/mmv-supported-projects

ii www.thesgc.org/sddc

iii www.thesgc.org/

## Glossary

Allele fraction: next-generation short-read sequencing returns the number of reads at a particular locus. The allele fraction represents the fraction of reads that are different from the reference genome
Cellular thermal-shift assay (CETSA): a label-free proteomics-based method that allows the quantification of a compound and its target interaction in intact cells
Copy number variation (CNV): the number of copies of a particular gene varies from one individual parasite to the next
EC_50_: the concentration of a drug that gives a half-maximal response
gRNA: a short guide (g)RNA that directs the Cas9 nuclease to bind and cut the target site in the genome
*In vitro* evolution and whole-genome analysis (IVIEWGA): a method used extensively with *P. falciparum* parasites for target discovery and chemical validation
Irresistible compound: a compound that, using *in vitro* evolution methods, failed to yield resistant parasites
Metabolic fingerprints (MetaPrints): a metabolomics approach to broadly characterize metabolic phenotypes and identify differences in specific parasite metabolites after a compound challenge
Minimum inoculum for resistance (MIR): the lower the MIR is, the higher the probability that *in vitro* drug resistance occurs
Mode of action (MOA): how a given drug exerts activity in a cell
Multidrug-resistance genes (MDRs): genes contributing to resistance mechanisms that render parasites resistant to multiple antimalarials independently of their mode of action
Single-nucleotide variant (SNV): a DNA sequence variation that occurs when a single nucleotide in the genome sequence is altered
Structure–activity relationship (SAR): a relationship that can be used to predict compound biological activities from their molecular structures
Target-less compound: a compound that, using *in vitro* evolution methods, yielded only general resistance mechanisms
